# Novel α-1,3-Glucosyltransferase Variants and Their Broad Clinical Polycystic Liver Disease Spectrum

**DOI:** 10.3390/genes14081652

**Published:** 2023-08-19

**Authors:** Melissa M. Boerrigter, René H. M. te Morsche, Hanka Venselaar, Nikki Pastoors, Anja M. Geerts, Anne Hoorens, Joost P. H. Drenth

**Affiliations:** 1 Department of Gastroenterology and Hepatology, Research Institute for Medical Innovation, Radboud University Medical Center, 6500 HB Nijmegen, The Netherlands; 2 Center for Molecular and Biomolecular Informatics, Research Institute for Medical Innovation, 6500 HB Nijmegen, The Netherlands; 3 Department of Gastroenterology and Hepatology, Ghent University Hospital, 9000 Ghent, Belgium; 4 Department of Pathology, Ghent University Hospital, 9000 Ghent, Belgium

**Keywords:** ADPLD, ALG8, clinical spectrum, next-generation sequencing, polycystic liver disease

## Abstract

Protein-truncating variants in α-1,3-glucosyltransferase (*ALG8*) are a risk factor for a mild cystic kidney disease phenotype. The association between these variants and liver cysts is limited. We aim to identify pathogenic *ALG8* variants in our cohort of autosomal dominant polycystic liver disease (ADPLD) individuals. In order to fine-map the phenotypical spectrum of pathogenic *ALG8* variant carriers, we performed targeted *ALG8* screening in 478 ADPLD singletons, and exome sequencing in 48 singletons and 4 patients from two large ADPLD families. Eight novel and one previously reported pathogenic variant in *ALG8* were discovered in sixteen patients. The *ALG8* clinical phenotype ranges from mild to severe polycystic liver disease, and from innumerable small to multiple large hepatic cysts. The presence of <5 renal cysts that do not affect renal function is common in this population. Three-dimensional homology modeling demonstrated that six variants cause a truncated ALG8 protein with abnormal functioning, and one variant is predicted to destabilize ALG8. For the seventh variant, immunostaining of the liver tissue showed a complete loss of ALG8 in the cystic cells. *ALG8*-associated ADPLD has a broad clinical spectrum, including the possibility of developing a small number of renal cysts. This broadens the ADPLD genotype–phenotype spectrum and narrows the gap between liver-specific ADPLD and kidney-specific ADPKD.

## 1. Introduction

Polycystic liver disease (PLD) is arbitrarily defined as the presence of >10 fluid-filled cysts in the liver [1]. In PLD, the hepatic function is preserved, but the increase in the number and size of liver cysts may compress the adjacent organs and lead to symptoms such as dyspnea, early satiety, and abdominal pain [1].

PLD is the most frequent extrarenal manifestation of autosomal dominant polycystic kidney disease (ADPKD). ADPKD is mainly caused by pathogenic variants in *PKD1* and *PKD2*, as shown in Table 1 [2,3]. The proteins encoded by these genes, PC1 and PC2, reside in the primary cilium [4,5,6]. Here, they are speculated to function as receptors or sensors, essential to various signaling pathways [4,7]. Both PC1 and PC2 also reside in the endoplasmic reticulum (ER), facilitating calcium release [6]. Defective calcium release from the ER is speculated to increase cyclic adenosine monophosphate (cAMP) levels, stimulating cystogenesis [5,6]. PLD is the primary clinical phenotype of autosomal dominant polycystic liver disease (ADPLD). ADPLD is caused by pathogenic variants in at least nine genes, as shown in Table 1. Most of these genes encode ER-resident enzymes that play a central role in the translocation of newly synthesized polypeptides into the ER, and are involved in the glycosylation, maturation, and quality control of nascent glycoproteins [2,3].

The exact pathophysiology of liver cyst development remains elusive [2,3,8,9,10,11]. The genetic underpinnings of PLD suggest the presence of a primary pathogenic germline variant in a PLD gene, and a secondary pathogenic somatic variant [1,3,9,12,13,14,15]. The consequences of these variants involve many molecular pathways, and a wide range of factors have been suggested including (but not limited to) cAMP, estrogen, primary cilia dysfunction, bile acid levels, cell–matrix remodeling, epigenetics, post-translational modifications, autophagy, and aberrant proteostasis [2,3,8,9,10,16,17,18,19,20,21,22,23,24,25,26]. This range of factors has resulted in a large variety in the potential targets for therapeutic treatment [2,3,8,9,11,16,17,18,19,20,21,22,23,24,25,26]. In addition, the genetic cause, in the majority of ADPLD individuals, is unknown [15]. The number of genetically diagnosed ADPLD individuals and the number of PLD-related genes increases yearly, due to the use of high-throughput screening techniques [27,28,29,30,31,32]. This will fuel a better understanding of PLD, and the identification of potential molecular pathways and therapeutic targets. *ALG8* encodes the similarly termed α-1,3-glucosyltransferase ALG8, an ER transmembrane protein that adds the second glucose group to lipid-linked oligosaccharides during N-linked glycosylation [33]. The loss of function in both *ALG8* alleles results in a specific congenital disorder of glycosylation (ALG8-CDG (OMIM #608104)) [34,35]. This severe form of CDG is characterized by manifestations such as facial dysmorphism, muscular hypotonia, hepatomegaly, coagulopathy (thrombocytopenia), gastrointestinal protein-losing enteropathy, edema, and ascites [34,35]. The loss of function in a single *ALG8* allele is associated with the development of hepatic cysts (ALG8-PLD (OMIM #617874)) [36].

The evaluation of the general population data indicates that pathogenic variants causing truncated ALG8 proteins are more prevalent than the number of diagnosed ADPLD patients with a heterozygous pathogenic *ALG8* variant [37,38]. A matched review of imaging found that individuals with a heterozygous variant causing truncated ALG8 are more likely to have cystic kidney disease (≥4 kidney cysts) (57.7% vs. 7.7%), but not liver cysts (11.5% vs. 7.7%) [38]. The lack of association between *ALG8* variants and liver cysts may arise from the composition of their cohort, as polycystic kidneys (1:1500) are more prevalent than polycystic livers (<1:10,000) in the general population [39,40]. In order to address the potential role of *ALG8* in cystic liver disease, we performed a comprehensive effort to discover pathogenic *ALG8* variants in a specific ADPLD population.

**Table 1:** Genes and proteins associated with PLD.

Gene		Protein		Associated Disease
Abbreviation	Full Name (NCBI ID)	Abbreviation	Full Name (UniProt ID)	
*ALG5*	ALG5 dolichyl-phosphate β-glucosyltransferase (29880)	ALG5	Dolichyl-phosphate β-glucosyltransferase (Q9Y673)	ADPKD [27]
*ALG8*	ALG8 α-1,3-glucosyltransferase (79053)	ALG8	Probable dolichyl pyrophosphate Glc1Man9GlcNAc2 α-1,3-glucosyltransferase (Q9BVK2)	ADPLD [36,38]
*ALG9*	ALG9 α-1,2-mannosyltransferase (79796)	ALG9	α-1,2-mannosyltransferase (Q9H6U8)	ADPKD and ADPLD [41,42,43]
*DNAJB11*	DnaJ heat shock protein family (Hsp40) member B11 (51726)	DJB11 or ERJ3	DnaJ homolog subfamily B member 11 (Q9UBS4)	ADPKD [44,45,46]
*GANAB*	Glucosidase II α subunit (23193)	GANAB or G2AN	Neutral α-glucosidase AB (Q14697)	ADPKD and ADPLD [36,47,48,49,50,51]
*IFT140*	Intraflagellar transport 140 (9742)	IF140	Intraflagellar transport protein 140 homolog (Q96RY7)	ADPKD [28]
*LRP5*	LDL receptor-related protein 5 (4041)	LRP5	Low-density lipoprotein receptor-related protein 5 (O75197)	ADPKD and ADPLD [52,53]
*PKD1*	Polycystin 1, transient receptor potential channel interacting (5310)	PC1 or PKD1	Polycystin-1 (P98161)	ADPKD [2,3,15]
*PKD2*	Polycystin 2, transient receptor potential cation channel (5311)	PC2 or PKD2	Polycystin-2 (Q13563)	ADPKD [2,3,15]
*PKHD1*	PKHD1 ciliary IPT domain containing fibrocystin/ polyductin (5314)	FC or PKHD1	Fibrocystin (P08F94)	ADPLD [36,54]
*PRKCSH*	Protein kinase C substrate 80K-H (5589)	GLU2B	Glucosidase 2 subunit β (P14314)	ADPLD [2,3,15]
*SEC61A1*	SEC61 translocon subunit α 1 (29927)	S61A1	Protein transport protein Sec61 subunit α isoform 1 (P61619)	ADPKD and ADPLD [29]
*SEC61B*	SEC61 translocon subunit β (10952)	SEC61β or SC61B	Protein transport protein Sec61 subunit β (P60468)	ADPLD [36]
*SEC63*	SEC63 homolog, protein translocation regulator (11231)	SEC63	Translocation protein SEC63 homolog (Q9UGP8)	ADPLD [2,3,15,51]

Gene abbreviation, full name, and ID from the National Center for Biotechnology Information (NCBI) [55]. Protein abbreviation, full name, and ID from UniProt [56]. PLD, polycystic liver disease; ADPKD, autosomal dominant polycystic kidney disease; ADPLD, autosomal dominant polycystic liver disease.

## 2. Material and Methods

### 2.1. Cohort Selection

ADPLD patients were included in our *ALG8* cohort based on the availability of biomaterials and clinical data in our PLD registry biobank [57]. This biobank includes patients diagnosed with PLD. PLD is defined as the presence of more than ten hepatic cysts detected via medical imaging (computed tomography, magnetic resonance imaging, or ultrasound) [1,57]. Patients were excluded from the cohort based on the criteria: ADPKD or diagnosed with a pathogenic variant in another PLD gene: *PRKCSH*, *SEC63*, *LRP5*, *GANAB*, *PKD1*, *PKD2*. ADPKD diagnosis was based on the Ravine criteria [58,59]. For all patients, genomic DNA was isolated from the available whole blood samples, according to the standard protocol of the High Pure PCR Template Preparation Kit (11796828001, Roche Life Science, Penzberg, Germany). The DNA concentration and quality (A260/A280 ratio: 1.80–1.99) were confirmed using the Infinite 200 Pro plate reader (Tecan, Männedorf, Switzerland) and the manufacturer’s quality guidelines.

### 2.2. Genetic Screening

Targeted sequencing of all individual *ALG8* exons (NM_024079.5), amplified via standard polymerase chain reaction (PCR), was performed using the MiniSeq Sequencing System (Illumina, San Diego, CA, USA). PCR was performed using a T100 Thermal Cycler (BioRad, Hercules, CA, USA). PCR protocol and primers (Sigma-Genosys, Haverhill, UK) are available in the Supplementary Material. Primers were designed to create fragments with a maximum of 300 nucleotides as, technically, from both sequencing directions, a maximum of 150 good-quality nucleotides can be read. Primers were designed using Primer3web software (version 4.1.0, Whitehead Institute for Biomedical Research, Cambridge, MA, USA), based on the reference genome: GRCh38 (hg38), with the gene ID of 79053, and the transcript ID of NM_024079.5 [60]. PCR products of all exons were diluted 1000-fold and combined per person. The combined PCR mixes were used for a second PCR with the same PCR reaction and protocol, to attach the individual-specific barcodes available in the Supplementary Material. Different combinations of forward and reverse barcode primers created individual-specific PCR mixes for 576 individuals. The PCR products for all individuals were pooled and purified using the Silica Bead DNA Gel Extraction Kit (K0513, Thermo Fisher Scientific, Waltham, MA, USA). Sequencing was performed in a single run on the MiniSeq system (Illumina), and data were analyzed with Sequence Pilot (version 5.1.0, JSI Medical Systems, Ettenheim, Germany). The selection criteria were: >10% variant variation to indicate heterozygous variants, >85% variant variation to indicate homozygous variants, non-synonymous variants, <0.001 minor allele frequency (MAF), deleterious by at least one prediction program (SIFT, MutationTaster, or PolyPhen-2), and exonic variant or intronic variant <10 base pairs from the splice site. No minimum reads were required. The four common single-nucleotide polymorphisms (SNPs) in *ALG8* (rs665278, rs6199592, rs17825668, and rs1263505) were excluded. Variants were confirmed according to the standard Sanger sequencing protocol with the Big Dye Terminator v1.1 Cycle Sequencing Kit (4337452, Thermo Fisher Scientific) and the 3730XL DNA Analyzer (Applied Biosystems, Waltham, MA, USA).

When available, family members were screened for the pathogenic variant via Sanger sequencing, as described above. In addition, some patients were identified via whole-exome sequencing (WES). DNA was enriched with the Twist Human Core Exome kit (104136, Twist Bioscience, South San Francisco, CA, USA), followed by 2 × 150 base paired-end sequencing on a NovaSeq 6000 Sequencing System (Illumina). Sequence reads were aligned to the GRCh37/hg19 human reference genome via the Burrows–Wheeler aligner [61]. Variant selection criteria were identical to the MiniSeq criteria described above. All *ALG8* variants called via WES were verified via Sanger sequencing, as described above.

The pathogenicity of the variants was determined using the American College of Medical Genetics and Genomics/American Association of Molecular Pathology (ACMG/AMP) classification guidelines [62], Alamut Visual Plus (version 1.4, SOPHiA GENETICS, Bidart, France), the human variation–phenotype archive ClinVar [63], and the population databases Genome Aggregation Database (gnomAD) and Exome Sequencing Project (ESP) Exome Variant Server, in June 2022 [62,64,65].

### 2.3. 3D Modeling

Three-dimensional structures were constructed based on the prediction of the human ALG8 (Q9BVK2) via AlphaFold DB (version 1 July 2021) (EMBL-EBI, Hinxton, UK) and the visualization program YASARA (YASARA Biosciences/ Bio-Prodict/ WHAT IF Foundation, Vienna, Austria and Nijmegen, the Netherlands) [66,67,68]. The schematic interpretation of the ALG8 protein and its transmembrane domains was based on 3D modeling analyses and the topological model of Albuquerque-Wendt et al. [69].

### 2.4. Immunohistochemistry Staining

Tissue sections (4 µm) on microscope slides (Thermo Fisher Scientific) were deparaffinized with xylene (JT Baker Avantor, Radnor, PA, USA) and ethanol (Merck, Darmstadt, Germany), and blocked with 3% endogenous hydrogen peroxide (Merck). The tissue was blocked using the Avidin/Biotin Blocking Kit (SP-2001, Vector Laboratories, Burlingame, CA, USA). Sections were incubated with 20% goat or horse serum (S-100/S-2000, Vector Laboratories), and incubated with the primary antibodies anti-ALG8 (rabbit, 1:50, HPA051898, Sigma-Aldrich, Burlington, MA, USA), anti-CK19 (mouse, 1:200, MU246-UC, BioGenex, Fremont, CA, USA), or anti-HNF4α (mouse, 1:200, Invitrogen MA1-199, Thermo Fisher Scientific) overnight at 4 °C. Tissue was incubated with the secondary biotinylated antibody goat anti-rabbit (1:200, BA-1000, Burlingame, CA, USA, Vector Laboratories) or horse anti-mouse (1:200, BA-2000, Vector Laboratories) for 30 min at room temperature. This was followed by incubation with avidin–biotin complex (ABC) (PK-6100, Vector Laboratories), 3,3′-diaminobenzidine (DAB) staining (1× (Sigma-Aldrich), 0.015% hydrogen peroxide (Merck)), Mayer’s hematoxylin staining (Sigma-Aldrich), and dehydration with ethanol and xylene. The protein expression and localization were visualized using the Zeiss Primovert microscope (Zeiss, Oberkochen, Germany), and analyzed using ZEN 2 blue (version 10, Zeiss).

## 3. Results

### 3.1. ALG8 Variants in the ADPLD Cohort

**Figure 1:**
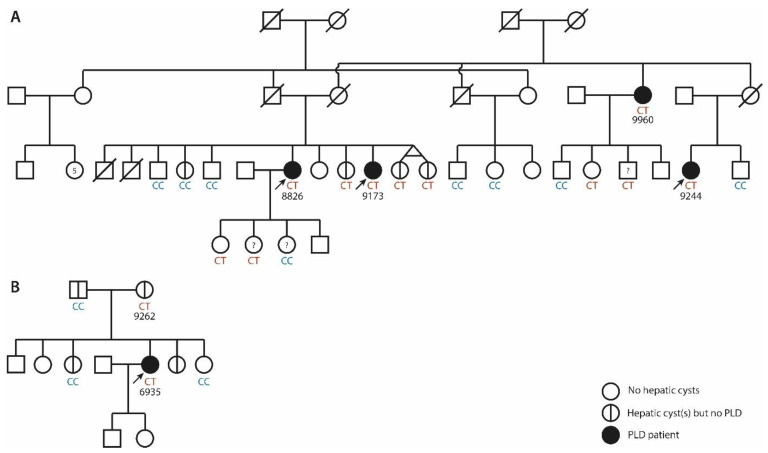
Pedigrees of the two ADPLD families with heterozygous *ALG8* variants. (**A**) Family 1 (c.160C>T p.(Gln54*)). (**B**) Family 2 (c.685C>T p.(Arg229*)). Circles and squares with a vertical stripe (|) indicate individuals with a small number of hepatic cysts who do not fulfill the ADPLD criteria (hepatic cyst(s) but no PLD). A diagonal stripe (/) indicates that this individual has passed away. Arrows indicate the individuals who have undergone exome sequencing. Blue indicates that the individual carries the two common nucleotides (CC), and red indicates that the individual carries one common nucleotide and one rare nucleotide (CT) at the genomic position of interest. In individuals without annotated CC or CT, the presence of hepatic cysts is unknown and no genetic screening has been performed. ?, these individuals have been genetically screened but the presence of hepatic cysts is unknown. 5, these parents have 6 children (one boy and 5 children of unknown sex).

Our ADPLD cohort consisted of 530 patients. Targeted *ALG8* screening was performed in 478 singletons, WES in 48 singletons, and WES in 4 ADPLD individuals from two families. From the overall group, 75.2% were female, and the average age was 56.8 years (ranging from 29 to 87 years). Using the MiniSeq system, we identified nine patients with a heterozygous and pathogenic *ALG8* variant, among our cohort of 478 ADPLD patients. A subsequent WES effort allowed us to discover two patients with pathogenic *ALG8* variants from a cohort of 48 singletons, and diagnose two ADPLD families where the *ALG8* variants segregated with the disease, as shown in Table 2 and Figure 1. These variants included four nonsense variants, three frameshift variants, one splice-site variant, and one missense variant. ClinVar reported two individuals with unknown conditions that carry the c.685C>T variant, one CDG individual with the c.981dupA variant, and 13 individuals with the c.1090C>T variant (two PLD, two CDG, and nine with unknown conditions). The c.1090C>T variant has been described in individuals with a small number of kidney cysts, with and without PLD [36,38]. The other eight variants have not been described before.

### 3.2. The Hepatic Phenotype of ALG8 Patients

#### 3.2.1. Family 1

**Figure 2:**
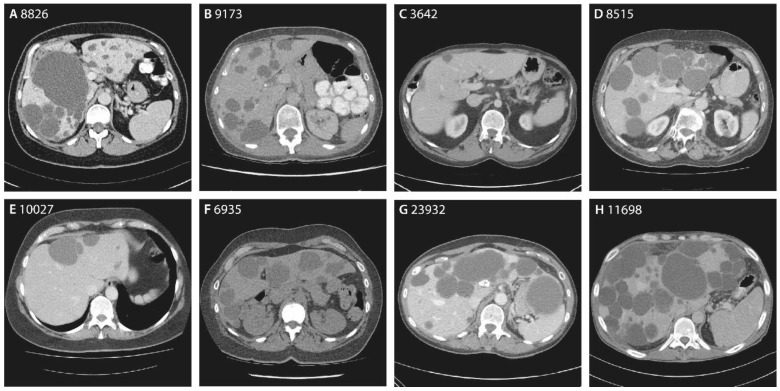
CT scans of eight ADPLD individuals with heterozygous pathogenic *ALG8* variants. (**A**) Individuals 8826 and (**B**) 9173 are part of family 1, and are carriers of the same nonsense variant (c.160C>T p.(Gln54*)). (**C**) Individuals 3642 and (**D**) 8515 are carriers of frameshift variants (c.272delA p.(Asn91Metfs*5) and c.371delG p.(Cys124Serfs*33)), (**E**) 10027 is a carrier of a splice-site variant (c.478+3A>G p.?), and (**F**) 6935, (**G**) 23932 and (**H**) 11698 are carriers of different nonsense variants (c.685C>T p.(Arg229*), c.1090C>T p.(Arg364*), and c.1501delG p.(Val501*)).

Family 1 is a large family, with clinical information and DNA from 19 family members. Four individuals are diagnosed with ADPLD, while four other individuals possess 1–2 hepatic cysts. Eight individuals did not have hepatic cysts at the time of screening, and the liver phenotype is unknown for the three remaining individuals; see Figure 1A. The index patient, 8826, has a liver phenotype with multiple small and medium-sized hepatic cysts (6–7 cm), and one very large cyst (12 cm); see Figure 2A. This has resulted in hepatomegaly, with a total liver volume (TLV) of 3780 mL (height-adjusted TLV: 2.32 L/m). Her sister, 9173, possesses numerous small, four medium-sized (5–6 cm), and one large (10 cm) cyst, Figure 2B. Their aunt, 9960, and their cousin, 9244, also have PLD. Several other family members were screened for liver cysts using ultrasound. Eight members have none, and four members have 1–2 hepatic cysts.

WES demonstrated the presence of the pathogenic nonsense variant c.160C>T p.(Gln54*) in this family. This heterozygous variant in *ALG8* was found in all four ADPLD individuals, three individuals with a small number of cysts in the liver, two individuals with an unknown clinical status, and two individuals with no cysts at the time of screening. The eight family members who do not carry this heterozygous variant include six individuals with no hepatic cysts, one individual with one hepatic cyst, and one individual who was not screened for hepatic cysts. The two family members with the heterozygous nonsense variant, but without hepatic cysts, were female, and 48 and 35 years old at the time of the ultrasound.

#### 3.2.2. Family 2

Family 2 is a six-sibling family, with one female, 6935, with more than 20 hepatic cysts of various sizes, Figure 1B. Her largest cysts are 7 and 12 cm, Figure 2F. Her PLD is mild and stable, with a liver volume of 2550 mL.

The heterozygous pathogenic *ALG8* variant c.685C>T p.(Arg229*) was found, using WES. Her mother also carries this heterozygous variant, and has three hepatic cysts. Her largest hepatic cyst is 2.9 cm. Her father does not carry a pathogenic variant in *ALG8* but has three hepatic cysts, the largest being 2.7 cm. Her older sister, who does not carry the nonsense variant, was diagnosed with five small hepatic cysts during screening for other health reasons. Her younger sister, who also does not carry the nonsense variant, showed no hepatic cysts in imaging that was performed for other health reasons.

#### 3.2.3. Singletons

Male 3642 was diagnosed with more than ten small hepatic cysts at a young age due to his prune belly syndrome; see Figure 2C. Male 24392 has a polycystic liver with at least ten larger hepatic cysts (1 cm) and two large cysts (more than 10 cm). He also has the heterozygous pathogenic variant c.685C>T p.(Arg229*), but is unrelated to family 2. Female 23932 has a polycystic liver with two large hepatic cysts (9 and 12 cm); see Figure 2G. Female 8094 has numerous hepatic cysts, with multiple large cysts up to 6 cm.

Male 762 was diagnosed with PLD at 66 years old. After the diagnosis, he never revisited our center for liver or kidney-related issues. At 87 years old, he received a CT scan for other health reasons. On this scan, he showed five hepatic cysts, which did not fulfill the PLD criteria.

Male 11698 used to have a polycystic liver, with cysts ranging from 1.5 to 9 cm. After partial liver resection combined with fenestration, he received liver–kidney transplantation. His kidneys were slightly atrophic and calcified, and contained a small number of renal cysts, ranging from 0.5 to 1.6 cm. His liver had a diameter of 17 × 28 × 10.5 cm, and weighed 2994 g. His liver showed signs of cyst ruptures and cyst bleeding, and his hepatic cysts were surrounded by fibrotic tissue; see Figure 2H.

Female 7906, male 8515 (Figure 2D), female 11549, female 10027 (Figure 2E), and female 11409 have ADPLD, but no further clinical information is available. Male 3642 and female 7906 are unrelated. Females 23932 and 11409 are unrelated. Male 11698 and female 8094 are unrelated.

### 3.3. Extrahepatic Manifestations in ALG8 Patients

For 11 out of the 16 patients, clinical information on the kidneys is available. Three individuals did not have renal cysts at the time of screening (6935, 23932, and 24392). The three patients with a pathogenic variant in the N-terminal side of ALG8 all had one small cyst on their left kidney (8826, 9173, and 8515). Patient 3642 had four bilateral kidney cysts. Patient 762 had two cysts in his left kidney, and one in his right kidney. Patient 10027 had one cyst in her left kidney. The two patients with the c.1501del variant had five and seven renal cysts (11698 and 8094). For patient 11698, the location of these cysts is unknown. Patient 8094 had four cysts in her left kidney, and three in her right kidney.

For 9 out of 16 patients, imaging was sufficient to determine abdominal wall hernias, a frequent extrahepatic manifestation in individuals with PLD [70]. Patients 9244 and 8515 had an umbilical hernia. The other seven patients did not have an abdominal wall hernia at the time of imaging (8826, 9173, 3642, 10027, 6935, 23932, and 11698).

### 3.4. Pathogenicity Prediction of ALG8 Variants

All nine *ALG8* variants are predicted to be either pathogenic, likely pathogenic, or variants of uncertain significance (VUSs), based on the ACMG/AMP guidelines. The three variants c.160C>T, c.371delG, and c.1501delG are not reported in the population databases gnomAD or ESP. The five variants c.460G>A, c.478+3A>G, c.685C>T, c.981dupA, and c.1090C>T are sporadically reported in these databases. In the world population, these variants have been detected 4, 5, 6, 25, and 18 times, respectively 0.00159%, 0.00199%, 0.00229%, 0.009944%, and 0.00637%. The gnomAD database does not report the frameshift variant c.272delA. However, the ESP database detected this variant 137 times in their population of 8583 European Americans (1.66%).

**Figure 3:**
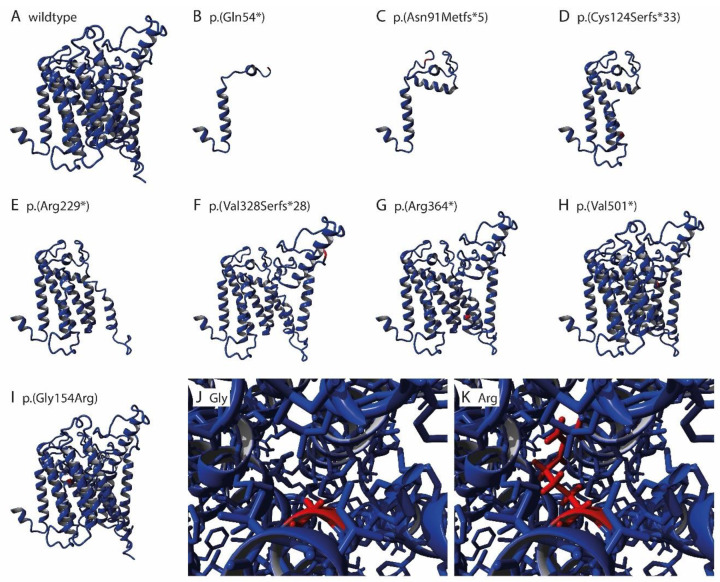

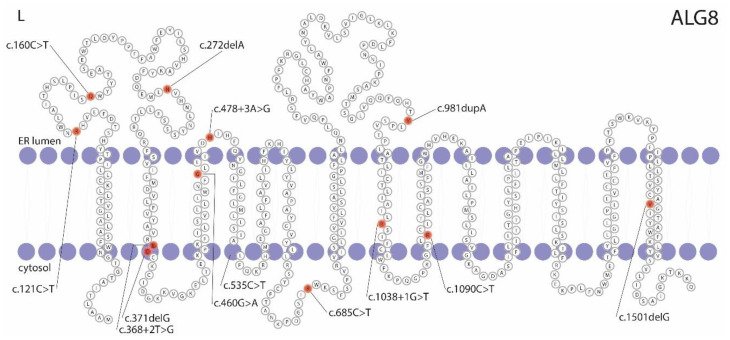
ALG8 protein and variant localizations. (**A**) 3D structure of the ALG8 wildtype; (**B**–**H**) 3D structure of the truncated ALG8 proteins due to the specified nonsense and frameshift variants; (**I**) 3D structure of ALG8 and the amino acid position 154 in red, with (**J**) a close up of the glycine side-chain at position 154, and (**K**) a close up of the arginine side-chain at position 154. (**L**) Schematic interpretation of the ALG8 protein, and the localization of pathogenic variants associated with liver and kidney cysts.

The 3D structure of the six nonsense and frameshift variants, p.(Gln54*), p.(Arg229*), p.(Arg364*), p.(Asn91Metfs*5), p.(Cys124Serfs*33), and p.(Val328Serfs*28) predicts the loss of numerous transmembrane α helices (Figure 3B–G). Thus, they will result in a non- or abnormal-functioning protein. The nonsense variant p.(Val501*) loses half of the last transmembrane helix (Figure 3H). It is uncertain if this will result in a normal functional protein, or if this alteration will affect the stability of the encoded ALG8 protein. The missense variant p.(Gly154Arg) is positioned in the core of the protein (Figure 3I–K). This variant changes the small and neutral amino acid glycine into the large, positively charged, and hydrophilic arginine. This change is predicted to affect the ALG8 folding, as the larger arginine side chain will destabilize the nearby transmembrane α helix. Further, the change in hydrophobicity will likely affect the hydrophobic interactions with the membrane lipids, and the difference in polarity can affect the ALG8 protein folding.

The splice-site variant c.478+3A>T alters the third nucleotide of intron 4. This position has a splicing preference for adenine or guanine. The alteration to a thymine is predicted to result in the potential loss of the exon 3 donor splice site. This altered splicing is predicted to result in the partial inclusion of intron 4 into the protein.

The eight novel variants, and the five previously reported variants [36,38], are positioned either at the N-terminal, in the middle, or at the C-terminal of the ALG8 protein (Figure 3L). They are located in the transmembrane regions, loops in the ER lumen, and loops in the cytosol region. Overall, the type of mutation or its location in the protein does not correlate with the clinical spectrum.

### 3.5. Somatic ALG8 Loss of Heterozygosity

**Figure 4:**
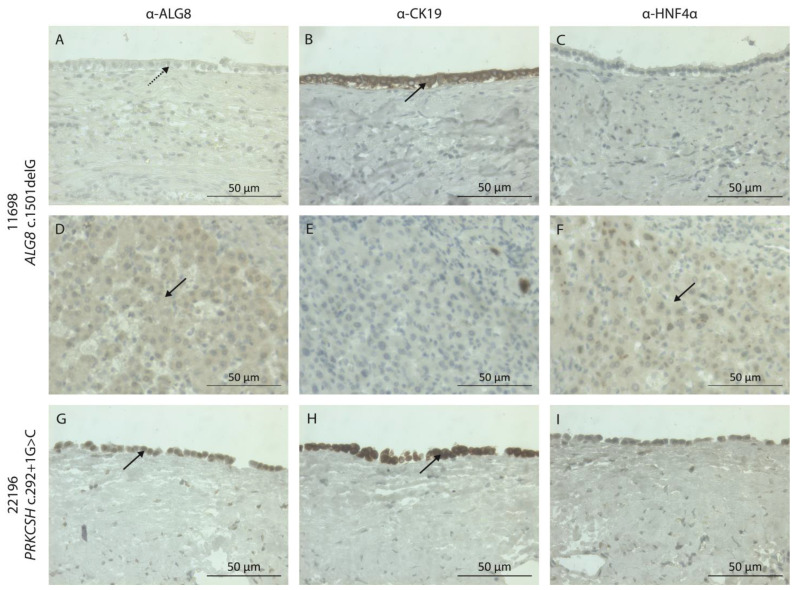
ALG8 expression in human liver tissue. (**A**–**F**) Individual 11698, with the *ALG8* nonsense variant c.1501delG p.(Val501*), and (**G**–**I**) an ADPLD individual with the *PRKCSH* splice-site variant c.292+1G>C p.?. (**A**,**D**,**G**) The protein of interest ALG8, (**B**,**E**,**H**) the cholangiocyte marker CK19, and (**C**,**F**,**I**) the hepatocyte marker HNF4α. Marker: 50 µm. The dotted arrow indicates the absence of marker staining. Solid arrows indicate the presence of marker staining. The key features of the liver cyst are displayed in Supplementary Figure S1.

Individual 11698, carrier of the heterozygous c.1501delG p.(Val501*) variant in *ALG8*, received liver transplantation. His cystic liver tissue was subjected to immunostaining for ALG8, cholangiocytes, and hepatocytes. The hepatic cyst lining originates from cholangiocytes, the epithelial cells of the bile duct. Therefore, a cholangiocyte marker is used to indicate the cystic lining. In patient 11698, the ALG8 protein is no longer present in the cholangiocytes or the cells bordering the cyst (Figure 4A,B). However, ALG8 is present in the hepatocytes more distant from the cystic lining (Figure 4D,F). In the cystic liver tissue of another ADPLD patient (*PRKCSH* c.292+1 p.?), ALG8 is expressed in the cholangiocytes, as expected (Figure 4G,H).

## 4. Discussion

In this study, we identified sixteen ADPLD patients and eight family members (with no, or a small number of, liver cysts) with heterozygous pathogenic variants in *ALG8*. We discovered eight unique, novel *ALG8* variants, and one previously reported variant (c.1090C>T p.(Arg364*)) [36,38]. The majority of these variants cause the premature termination of the translated ALG8 peptide, and result in an incomplete protein. In contrast, *ALG8* missense variants in our ADPLD population are uncommon, but drastically affect protein folding, and likely alter ALG8 functioning.

The clinical spectrum of ADPLD is broad in individuals possessing a heterozygous pathogenic *ALG8* variant. The phenotype ranges from a few liver cysts to severe PLD. The c.1090C>T p.(Arg364*) variant is seen in relation to a broad clinical spectrum: PLD without renal cysts (this study), PLD with 1–9 renal cysts (this study) [36,38,71], 0–1 hepatic cyst with ≥4 renal cysts [38], or no hepatic and no renal cysts [38]. A small number of renal cysts is common in this population, which is atypical in *PRKCSH*- and *SEC63*-caused ADPLD. The presence of these renal cysts does not affect the renal function, or cause end-stage renal disease, as seen in *PKD1*- and *PKD2*-caused ADPKD. Males are more prevalent in this group (31% (5/16)) than in the general ADPLD population (15–20%) [1,57]. Overall, *ALG8*-associated PLD possesses the ADPLD phenotype with hepatic cysts, but patients may also develop renal cysts, which can mimic early-stage ADPKD. However, clinically relevant renal function decline and renal failure are not reported in our population. This *ALG8*-phenotype narrows the current gap between ADPLD with a restricted liver phenotype and ADPKD.

Liver cysts are not exclusively caused by *ALG8*. Other heterozygous pathogenic variants in N-linked glycosylation genes have also been associated with kidney and liver cysts. Indeed, *ALG5* is found in the ADPKD population without PLD, and *ALG9* in individuals with ADPKD who also have liver cysts [27,41]. Additionally, *ALG8* has also been identified in cystic kidney disease (≥4 kidney cysts) without any PLD connection, and in ADPLD with multiple kidney cysts [36,38]. The functionality, maturation, and localization of polycystin-1 has been shown to be impacted by the inactivation of these genes [27,36,41]. Everything indicates that these enzymes, essential for peptide glycosylation in the ER, have a negative effect on the major ADPKD protein. This implies that, even though the dysfunction of these enzymes impacts the glycosylation of a wide variety of proteins, the kidney and liver cysts in these individuals emerge and develop through a similar molecular mechanism as *PKD1*-caused ADPKD with PLD.

The molecular mechanism underpinning polycystic liver development is unclear, but a somatic second hit has been suggested to play a role [1,12,13]. In this model, individuals who carry a heterozygous pathogenic germline variant (first hit) are prone to develop a disease stemming from molecular events associated with that gene. Specifically, cellular recessiveness and somatic loss of heterozygosity (LOH) occur when an additional pathogenic somatic variant (second hit) causes the loss of both wildtype alleles. A second somatic point mutation or a large LOH region and the complete absence of a PLD protein have been observed in cystic livers from ADPLD and ADPKD patients carrying a heterozygous pathogenic germline variant [12,72,73]. One of our severely affected ADPLD patients was the carrier of a heterozygous germline variant in *ALG8* (c.1501delG). The immunohistochemistry of his liver cyst tissue demonstrated the complete loss of the ALG8 protein in the cyst wall. Unfortunately, as a result of the sample’s poor quality, it was impossible to extract DNA to confirm this on a molecular level. Nevertheless, the results suggest the hepatic cyst formation results from LOH, and supports the somatic second hit hypothesis.

Large population-database screening has demonstrated that protein-truncating variants in ADPLD-associated genes are present in 0.202% of the general population [37]. Within this group of ADPLD genes, protein-truncating variants in *PRKCSH*, *SEC63*, and *ALG8* account for 23.8%, 10.4%, and 34.7%, respectively [37]. This implies that *ALG8* should be a major ADPLD-causing gene, and should appear more often in the ADPLD population than is currently reported. Our cohort contained more ADPLD individuals with a pathogenic *ALG8* variant than expected, based on previous reports, but no more than the number of ADPLD individuals with a pathogenic *PRKCSH* variant in our PLD registry [1,74]. This suggests that there are individuals with asymptomatic *ALG8*-caused ADPLD in the general population, or that *ALG8*-caused ADPLD has a milder phenotype.

The first suggestion relates to incomplete penetrance. If ADPLD genes showed complete penetrance, the ADPLD prevalence would be in the range of 1 in 46 to 496 individuals [37]. However, the prevalence of isolated polycystic livers in cohorts from the general population has been detected as much lower [40,75]. This suggests that a pathogenic variant in an ADPLD gene does not necessarily result in the development of a polycystic liver, and that incomplete penetrance plays a role in ADPLD.

The second suggestion, genetic expressivity, is the degree of inter-individual variability in a phenotype. Due to a high inter-individual variability, genotype–phenotype correlations are limited in the ADPLD population [3,36,74]. This high inter-individual variability is also seen in our *ALG8*-caused ADPLD population. Unrelated individuals, or even family members, with the same pathogenic *ALG8* variant do not have a similar degree of ADPLD severity, and some only have a small number of hepatic cysts. This broad variation implicates the involvement of genetic modifiers or environmental factors associated with variable expressivity. One of these risk factors is speculated to be estrogen-containing oral contraceptives [18,76]. This suggests that genetic expressivity plays a role in ADPLD, and explains the high inter-individual variability observed in ADPLD patients with a heterozygous pathogenic *ALG8* variant.

Our results corroborate the concept that ADPLD is caused by genes encoding ER-resident proteins. The ALG8 enzyme is essential in N-linked glycosylation, a co-translational modification step in the ER that promotes proper protein folding [33]. Missense variants in N-linked glycosylation enzymes can result in large structural and functional changes to the protein and, as a result, affect the complete N-linked glycosylation process [77]. Defective N-linked glycosylation results in misfolded proteins subjected to degradation to prevent ER stress [78]. When insufficient degradation leads to chronic high or low ER stress levels, this results in cellular apoptosis or proliferation, respectively [25]. Apoptosis is critical in liver fibrosis and cirrhosis development, a phenotype seen in homozygous ALG8-CDG [79]. Cellular proliferation is one of the hallmarks of hepatic cystogenesis, the phenotype of heterozygous ALG8-ADPLD [25]. This supports the concept that the dysfunction of various ER proteins can cause ADPLD development.

The strength of this study is the availability of a large cohort of well-characterized ADPLD individuals. However, as the study is based at an expertise center, this cohort mainly consists of individuals with severe PLD or serious symptoms. Consequently, individuals with asymptomatic or mild ADPLD are a minority in this cohort, which could result in a biased clinical phenotype and prevalence. The screening of cohorts containing more mild-ADPLD individuals will clarify if the broad phenotypic spectrum is coherent with the whole ADPLD population. Another limitation is our technique, which only screens for pathogenic variants in exons and splice-site regions. This excludes (deep) intronic variants in the non-coding regions. The predictive knowledge on the effect of these variants is limited, but the number of deep intronic variants explaining a wide variety of human diseases is increasing [80]. Deep intronic variants have been determined to cause sporadic cases of recessive glycosylation disorders and recessive PKD [81,82,83,84]. Patients with unexplained ADPLD may possess (deep) intronic *ALG8* variants.

In conclusion, pathogenic variants in *ALG8* are also associated with ADPLD, and those affected possess no or only a few kidney cysts. Our results support the concept of LOH, and the somatic second hit hypothesis, in PLD.

## Figures and Tables

**Table 2:** Molecular and clinical information of 16 ADPLD individuals with heterozygous pathogenic *ALG8* variants.

Chromosome Position	Nucleotide Change	Amino Acid Change	Variant Type	ACMG/AMP	Patient	Sex	Age	Hepatic Cysts	Imaging	GGT	Renal Cysts	eGFR	
g.78127372G>A	c.160C>T	p.(Gln54*)	Nonsense	Pathogenic	9960	Female	87	PLD	US	-	-	-	Family 1
					8826	Female	60	20+	CT	44	1	69	
					9173	Female	44	20+	CT	15	1	84	
					9244	Female	58	PLD	US	-	-	-	
g.78124118del	c.272delA	p.(Asn91Metfs*5)	Frameshift	VUS	3642	Male	29	10+	CT	17	4	89	
					7906	Female	50	PLD	US	-	-	-	
g.78121172del	c.371delG	p.(Cys124Serfs*33)	Frameshift	Likely pathogenic	8515	Male	75	20+	CT	762 *^a^*	1	>90	
g.78121083C>T	c.460G>A	p.(Gly154Arg)	Missense	VUS	11549	Female	73	PLD	US	-	-	-	
g.78121062T>C	c.478+3A>G	p.?	Splice-site	VUS	10027	Female	56	10+	CT	29	1	77	
g.78113978G>A	c.685C>T	p.(Arg229*)	Nonsense	Pathogenic	6935	Female	48	20+	CT	16	0	84	Family 2
					24392	Male	68	PLD	US	-	0	86	
g.78109499dup	c.981dupA	p.(Val328Serfs*28)	Frameshift	Pathogenic	762	Male	66	5	CT	25	3	79	
g.78106895G>A	c.1090C>T [36,38]	p.(Arg364*)	Nonsense	Pathogenic	23932	Female	59	20+	CT	410 *^a^*	0	>90	
					11409	Female	86	PLD	US	-	-	-	
g.78101044del	c.1501delG	p.(Val501*)	Nonsense	Likely pathogenic	11698	Male	57	20+	CT	-	5	-	
					8094	Female	48	PLD	US	-	7	-	

Chromosome position is based on GRCh38/hg38. The coding DNA position is based on NM_024079.5. VUS: variant of uncertain significance. -: data are not available. US: ultrasound scan, CT: computed tomography scan. PLD: polycystic liver disease with more than ten liver cysts, but where ultrasound scans could not determine the exact number of liver cysts. GGT: γ-glutamyl transferase, reference value male: <50, female: <40. eGFR: estimated glomerular filtration rate, reference value: >60 mL/min/1.73 m^2^. p.?, effect on protein level is expected but reliable prediction of the consequences is not possible. *^a^* individuals 8515 and 23932 had a (hepatic cyst) infection during blood diagnostics.

## Data Availability

Additional data are available from the corresponding author upon request.

## Supplementary Materials

The following supporting information can be downloaded at: https://www.mdpi.com/article/10.3390/genes14081652/s1, Figure S1: Liver cyst key features.
