# Supplementary material for: Novel α-1,3-Glucosyltransferase Variants and Their Broad Clinical Polycystic Liver Disease Spectrum
Source: Genes (Basel). 2023 Aug 19;14(8):1652. doi: 10.3390/genes14081652 (PMC10454741; doi:10.3390/genes14081652)
Supplement: Supplementary file 1 [file genes-14-01652-s001.zip › genes-2465933-supplementary.pdf]

Novel *ALG8* variants and their broad clinical polycystic liver disease spectrum
Boerrigter et al. (2023)[43]

Supplementary material

MiniSeq preparation protocol

95 °C	3 minutes	
95 °C	30 seconds	40 cycles
60 °C	30 seconds	40 cycles
72 °C	30 seconds	40 cycles
72 °C	5 minutes	

Primers for amplification of *ALG8* exons (NM_024079.5)

Exon & direction	Primer
1-F	GTAATCGGGAAGCTGAAGCGGCTTCAGAGGGCTTTCT
1-R	GATCCGACGGTAGTGTATCCACACCTTTCTCTCCCG
2-F	GTAATCGGGAAGCTGAAGCTGTGACAGCTTAGGGAATGT
2-R	GATCCGACGGTAGTGTCACCCAGCCAGAAAACATTT
3-F	GTAATCGGGAAGCTGAAGTCGTTGCACCATCTTTGTTTATT
3-R	GATCCGACGGTAGTGTTCCATACAAAATGACATGCTCCA
4-F	GTAATCGGGAAGCTGAAGTCCGATGAAGATCAAAGTTGTTT
4-R	GATCCGACGGTAGTGTTCAGAAGGAGCTCACATCTC
5-F	GTAATCGGGAAGCTGAAGGAGCAGTCCACATTAGTAGTAGT
5-R	GATCCGACGGTAGTGTACCCACTACACTGTTCCCTTT
6-F	GTAATCGGGAAGCTGAAGAATGTCCTTGTTCTGTTCCAG
6-R	GATCCGACGGTAGTGTACCAAGTCAACACAGTAAGGG
7-F	GTAATCGGGAAGCTGAAGTGGTTAATGTTCCTTTCCATCTT
7-R	GATCCGACGGTAGTGTGGGGAGTCACGTTGGGTAA
8-F	GTAATCGGGAAGCTGAAGACCATAGAGAGTTCCCTAGTTCT
8-R	GATCCGACGGTAGTGTTTCCATTTCTCCATGTGCCT
9-F	GTAATCGGGAAGCTGAAGCCTTTAAGCAGCAATAAGATTT
9-R	GATCCGACGGTAGTGTTGAGCACCATCTGTTGAGTTC
10-F	GTAATCGGGAAGCTGAAGTTCACCTGAACTCTGTTTCTGT
10-R	GATCCGACGGTAGTGTCCAAAATGCTCACTGGCTGA
11-F	GTAATCGGGAAGCTGAAGGCCTCAGCCAGTTCTAATGTAAT
11-R	GATCCGACGGTAGTGTATCTGTGGGCCTCGATGAAA
12-F	GTAATCGGGAAGCTGAAGCAGCATCATAAGTCTTTCTGTCA
12-R	GATCCGACGGTAGTGTGTGATTTCTCCCTCTATTTACCA
13a-F	GTAATCGGGAAGCTGAAGTTGACTCTGGCCTTGGCTAT
13a-R	GATCCGACGGTAGTGTCTGCACAATACACTGAGGTTAGT
13b-F	GTAATCGGGAAGCTGAAGTGGATGGAAACTTTCTACCTGC
13b-R	GATCCGACGGTAGTGTTCCCATGAAATAATTGCTTGGC

F: Forward primer, R: Reverse primer

Barcodes for MiniSeq preparation

Number & direction	Primer
001-F	AATGATACGGCGACCACCGAGATCTACACACGGAACCTGATACGAGATCCGTAATCGGGAAGCTGAAG

Novel *ALG8* variants and their broad clinical polycystic liver disease spectrum
Boerrigter et al. (2023)[43]

009-F	AATGATACGGCGACCACCGAGATCTACACAATCCGTATGATACGAGATCCGTAATCGGGAAGCTGAAG
017-F	AATGATACGGCGACCACCGAGATCTACACCGAATAGATGATACGAGATCCGTAATCGGGAAGCTGAAG
097-F	AATGATACGGCGACCACCGAGATCTACACCCATGATTCGATACGAGATCCGTAATCGGGAAGCTGAAG
105-F	AATGATACGGCGACCACCGAGATCTACACAGAATCCTCGATACGAGATCCGTAATCGGGAAGCTGAAG
113-F	AATGATACGGCGACCACCGAGATCTACACTGGCGTTGCGATACGAGATCCGTAATCGGGAAGCTGAAG
193-F	AATGATACGGCGACCACCGAGATCTACACCTTGCAGTAGATACGAGATCCGTAATCGGGAAGCTGAAG
201-F	AATGATACGGCGACCACCGAGATCTACACGACGGAATAGATACGAGATCCGTAATCGGGAAGCTGAAG
209-F	AATGATACGGCGACCACCGAGATCTACACTTGGTTCGAGATACGAGATCCGTAATCGGGAAGCTGAAG
289-F	AATGATACGGCGACCACCGAGATCTACACGTACCAATTCATACGAGATCCGTAATCGGGAAGCTGAAG
297-F	AATGATACGGCGACCACCGAGATCTACACTACTTCCGTCATACGAGATCCGTAATCGGGAAGCTGAAG
305-F	AATGATACGGCGACCACCGAGATCTACACTCGGAGAGTCATACGAGATCCGTAATCGGGAAGCTGAAG
385-F	AATGATACGGCGACCACCGAGATCTACACGTTATTGGTTATACGAGATCCGTAATCGGGAAGCTGAAG
393-F	AATGATACGGCGACCACCGAGATCTACACCCATTGCGTTATACGAGATCCGTAATCGGGAAGCTGAAG
401-F	AATGATACGGCGACCACCGAGATCTACACGCCAATAGTTATACGAGATCCGTAATCGGGAAGCTGAAG
481-F	AATGATACGGCGACCACCGAGATCTACACTTCTTCCGGTATACGAGATCCGTAATCGGGAAGCTGAAG
489-F	AATGATACGGCGACCACCGAGATCTACACTCCAGAAGGTATACGAGATCCGTAATCGGGAAGCTGAAG
497-F	AATGATACGGCGACCACCGAGATCTACACTGAGTTGCGTATACGAGATCCGTAATCGGGAAGCTGAAG
577-F	AATGATACGGCGACCACCGAGATCTACACGATAGTCGCTATACGAGATCCGTAATCGGGAAGCTGAAG
585-F	AATGATACGGCGACCACCGAGATCTACACCGAATGAGCTATACGAGATCCGTAATCGGGAAGCTGAAG
593-F	AATGATACGGCGACCACCGAGATCTACACGTTGAGTCCTATACGAGATCCGTAATCGGGAAGCTGAAG
673-F	AATGATACGGCGACCACCGAGATCTACACAATTGGCGATATACGAGATCCGTAATCGGGAAGCTGAAG
681-F	AATGATACGGCGACCACCGAGATCTACACCTAACTAGATATACGAGATCCGTAATCGGGAAGCTGAAG
689-F	AATGATACGGCGACCACCGAGATCTACACTAGCCGTCATATACGAGATCCGTAATCGGGAAGCTGAAG
001-R	CAAGCAGAAGACGGCATACGAGATGTTATTGGTTACACGCACGATCCGACGGTAGTGT
009-R	CAAGCAGAAGACGGCATACGAGATCCATTGCGTTACACGCACGATCCGACGGTAGTGT
017-R	CAAGCAGAAGACGGCATACGAGATGCCAATAGTTACACGCACGATCCGACGGTAGTGT
097-R	CAAGCAGAAGACGGCATACGAGATTTCTTCCGGTACACGCACGATCCGACGGTAGTGT
105-R	CAAGCAGAAGACGGCATACGAGATTCCAGAAGGTACACGCACGATCCGACGGTAGTGT
113-R	CAAGCAGAAGACGGCATACGAGATTGAGTTGCGTACACGCACGATCCGACGGTAGTGT
193-R	CAAGCAGAAGACGGCATACGAGATGATAGTCGCTACACGCACGATCCGACGGTAGTGT
201-R	CAAGCAGAAGACGGCATACGAGATCGAATGAGCTACACGCACGATCCGACGGTAGTGT
209-R	CAAGCAGAAGACGGCATACGAGATGTTGAGTCCTACACGCACGATCCGACGGTAGTGT
289-R	CAAGCAGAAGACGGCATACGAGATAATTGGCGATACACGCACGATCCGACGGTAGTGT
297-R	CAAGCAGAAGACGGCATACGAGATCTAACTAGATACACGCACGATCCGACGGTAGTGT
305-R	CAAGCAGAAGACGGCATACGAGATTAGCCGTCATACACGCACGATCCGACGGTAGTGT
385-R	CAAGCAGAAGACGGCATACGAGATACGGAACCTGACACGCACGATCCGACGGTAGTGT
393-R	CAAGCAGAAGACGGCATACGAGATAATCCGTATGACACGCACGATCCGACGGTAGTGT
401-R	CAAGCAGAAGACGGCATACGAGATCGAATAGATGACACGCACGATCCGACGGTAGTGT
481-R	CAAGCAGAAGACGGCATACGAGATCCATGATTCGACACGCACGATCCGACGGTAGTGT
489-R	CAAGCAGAAGACGGCATACGAGATAGAATCCTCGACACGCACGATCCGACGGTAGTGT
497-R	CAAGCAGAAGACGGCATACGAGATTGGCGTTGCGACACGCACGATCCGACGGTAGTGT
577-R	CAAGCAGAAGACGGCATACGAGATCTTGCAGTAGACACGCACGATCCGACGGTAGTGT
585-R	CAAGCAGAAGACGGCATACGAGATGACGGAATAGACACGCACGATCCGACGGTAGTGT
593-R	CAAGCAGAAGACGGCATACGAGATTTGGTTCGAGACACGCACGATCCGACGGTAGTGT
673-R	CAAGCAGAAGACGGCATACGAGATGTACCAATTCACACGCACGATCCGACGGTAGTGT
681-R	CAAGCAGAAGACGGCATACGAGATTACTTCCGTCACACGCACGATCCGACGGTAGTGT
689-R	CAAGCAGAAGACGGCATACGAGATTCGGAGAGTCACACGCACGATCCGACGGTAGTGT

F: Forward primer, R: Reverse primer

Novel *ALG8* variants and their broad clinical polycystic liver disease spectrum
Boerrigter et al. (2023)[43]

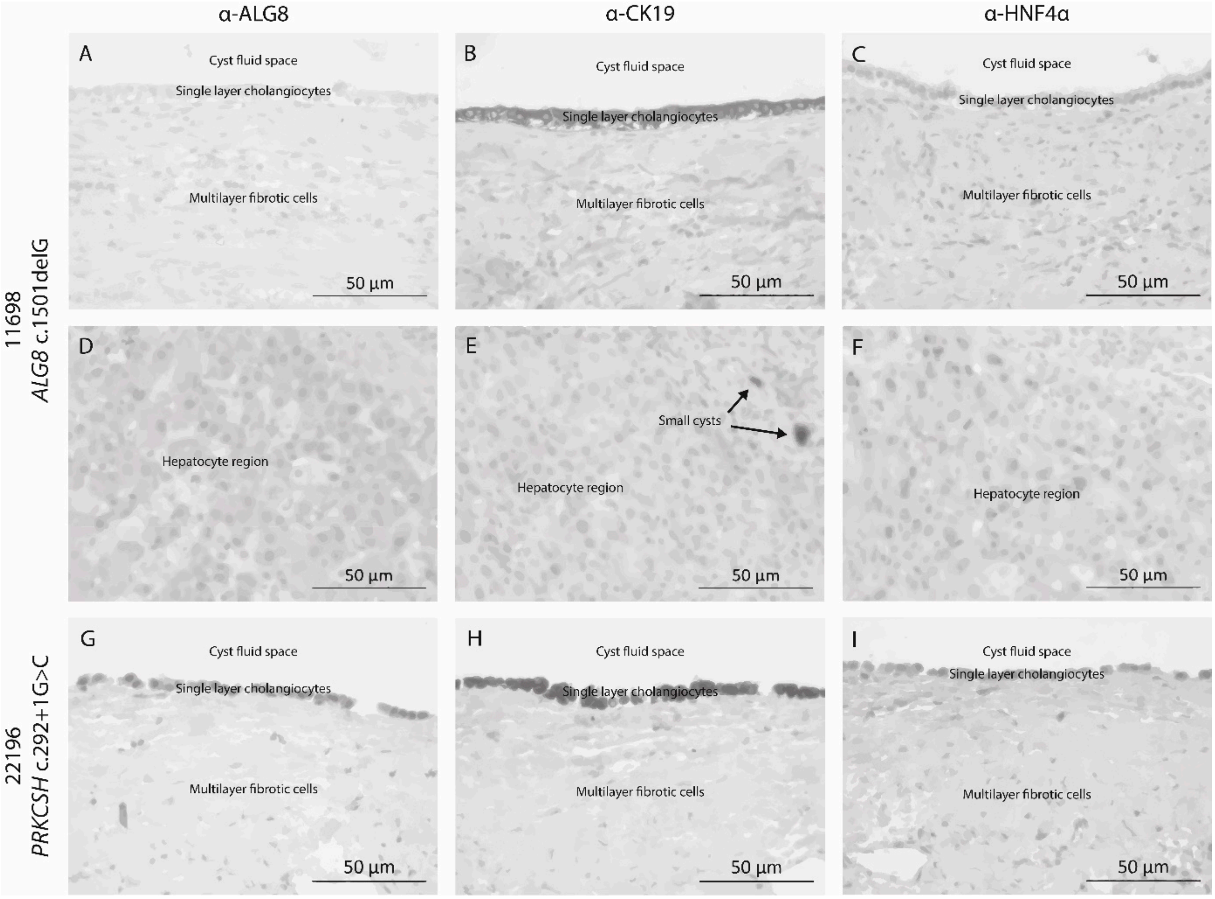

Supplementary figure S1 Liver cyst key features. **A-C** and **G-I** Large cysts are encapsulated by a single layer of cholangiocytes followed my multiple layers of fibrotic/connective tissue. Lobules, portal veins, hepatic arteries, and hepatocytes are absent. **D-F** Hepatocytes remain in less severely affected regions. However, lobules, portal veins, and hepatic arteries are severely diminished. Small liver cysts start to arise (**E**).
